# Not from the Stars Do I My Judgment Pluck^1^

**DOI:** 10.3201/eid1608.AC1608

**Published:** 2010-08

**Authors:** Polyxeni Potter

**Affiliations:** Centers for Disease Control and Prevention, Atlanta, Georgia, USA

**Keywords:** Art science connection, emerging infectious diseases, art and medicine, Vincent van Gogh, Terrace of a Café at Night, influenza, stars, about the cover

**Figure:**
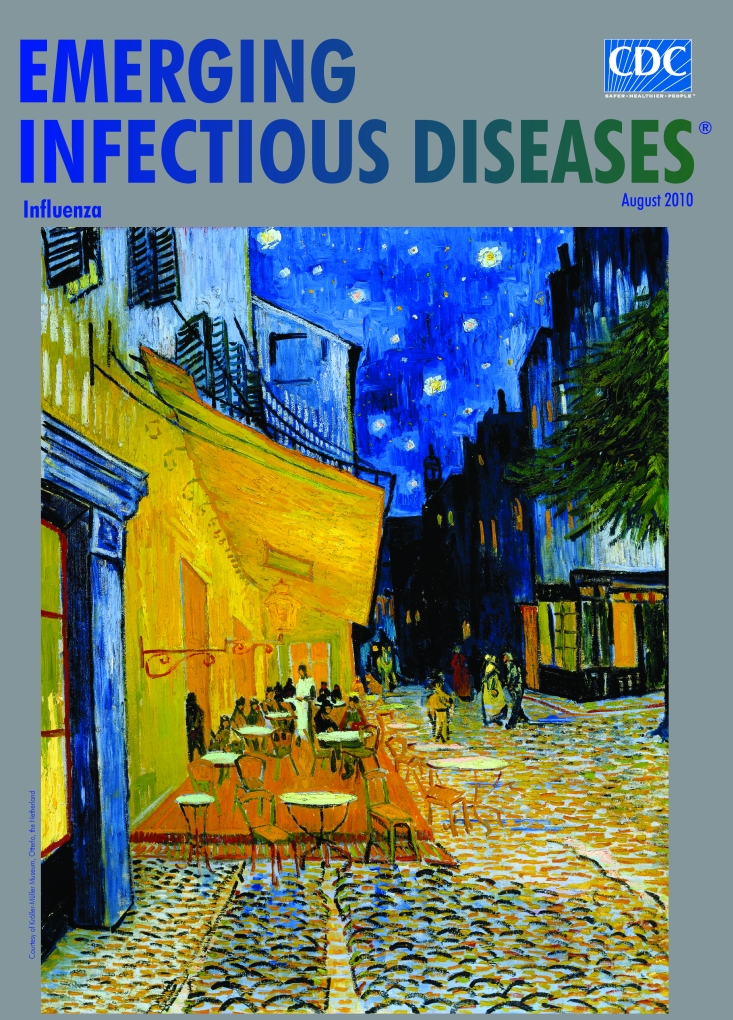
**Vincent van Gogh (1853–1890) *Terrace of a Café at Night (Place du Forum)* (c. 18 September 1888)** Oil on canvas (80.7 cm × 65.3 cm) Courtesy of Kröller-Müller Museum, Otterlo, the Netherlands

“It amuses me enormously to paint the night right on the spot,” wrote Vincent van Gogh to his brother Theo. “Normally, one draws and paints the painting during the daytime after the sketch. But I like to paint the thing immediately. It is true that in the darkness I can take a blue for a green, a blue lilac for a pink lilac, since it is hard to distinguish the quality of the tone. But it is the only way to get away from our conventional night with poor pale whitish light ….” Despite this affection for the night, van Gogh described *Night Café,* one of his best known night paintings, as “one of the ugliest I have done.” Though he loved the purity of the night outdoors, he loathed urban night life. “I have attempted to show that the café is a place where a man can ruin himself, become mad, commit a crime….” He moved away from Paris, where he lived with Theo, to Arles, “wishing to see a new light” and explore the calm.

In Paris he had come to know the impressionists and to experiment with broken brushstrokes and the style of the pointillists Georges Seurat and Paul Signac. He studied with Fernand Cormon and made friendships and contacts in the art world. His palette was transformed, from dark tones and stillness to yellows and blues and swirling lines. Yet, “When I left you at the station to go south,” he told Theo, “I was very miserable, almost an invalid and almost a drunkard. Now at last something is beginning to show on the horizon: Hope.” Moving to the countryside was an effort to get in touch with a more authentic way to live, to focus on ideas and nourish the spirituality he long sought, first as a student at the seminary and then in art.

The simplicity of rural life appealed to him on another level. “I will begin by telling you that this country seems to me as beautiful as Japan as far as the limpidity of the atmosphere and the gay color effects are concerned.” Like many of his contemporaries, van Gogh was fascinated with art from the Orient. He collected and copied woodblock prints and welcomed Utagawa Hiroshige and Katsushika Hokusai into the western vernacular. “My whole work … builds so to speak on what the Japanese have done.” Under their influence, he moved toward color and away from naturalism, volume and perspective, light and shadow. “I envy the Japanese artists for the incredible neat clarity which all their works have. It is never boring and you never get the impression that they work in a hurry. It is as simple as breathing; they draw a figure with a couple of strokes … as if it were as easy as buttoning one’s waistcoat.”

Van Gogh’s meteoric rise to greatness in the so-brief span of his 37 years took place in various settings and was marked by emotional turmoil, from unrequited love and failure at evangelism to familial strife and poverty. Through it all, he assessed his own legacy as “of very secondary importance.” Largely self-taught, he absorbed brief but potent influences. He took his first artistic steps in his native Holland, copying from art books, working as apprentice for an art dealer at age 16. He received formal instruction from leading Hague School artist Anton Mauve, then moved to London, where he taught school for a couple of years. He became interested in the Barbizon group, particularly Jean-François Millet, and started to paint peasants and rural life, a practice he would continue throughout his life. He traveled to Belgium to study at the Antwerp Academy, an unsuccessful venture, and soon after went to live with Theo in Paris. He took up painting in earnest in 1880 and continued until his death, producing in 10 years 900 paintings and more than 1,100 works on paper. Some of his masterpieces were created during the past 2 years of life when, overcome by mental illness, he committed himself to the asylum in Saint-Rémy. “I put my heart and my soul into my work and have lost my mind in the process.”

The evening and night, recurring themes in van Gogh’s work, interested him even before he began to paint. As a youth he was an avid reader, fluent in Dutch, German, English, and French. Many of the books he mentioned in his letters described the spiritual and poetic character of the night, the interval between sunset and dark, and the darkness between dusk and dawn. “It seems to me that the night is more alive and richly colored than the day.” This time for reflection and introspection sparked his artistic imagination and produced, among other major works, *The Starry Night; Landscapes at Twilight; Peasant Life at Evening; Poetry of the Night;* and *Terrace of a Café at Night*, on this month’s cover, a painting reminiscent of Hiroshige’s *Scene of the Saruwaka-cho Theater Street by Night.*

“On the terrace there are small figures of people drinking,” van Gogh wrote to his brother about this his first starry painting of an outdoor café. “An immense yellow lantern illuminates the terrace, the facade, the sidewalk, and even casts light on the paving stones of the road, which take a pinkish violet tone. The gables of the houses, like a fading road below a blue sky studded with stars, are dark blue or violet with a green tree.” Excited about the results, he explained to Theo, “Here you have a night painting without black, with nothing but beautiful blue and violet and green and in this surrounding the illuminated area colors itself sulfur pale yellow and citron green.”

In this and other night paintings, he struggled to achieve luminosity with contrasting or exaggerated colors and to demonstrate the superiority of natural light and the imagination over artificial light and reality. He struggled equally to express the mysterious influence of the night on the human heart as he understood it from his own tumultuous life. “I am a man of passion, capable and prone to undertake more or less foolish things which I happen to repent more or less.” While he worked on his first painting of a starry night, he wrote, “It is good for me to work hard. But that does not keep me from having a terrible need of―shall I say the word―yes, of religion. Then I go out at night to paint the stars.”

This need went back to van Gogh’s days as evangelist in an impoverished mining town in Belgium. He was dismissed from that post for showing extreme charity and identifying too much with the flock. His religious zeal dampened, he vowed then to make art for the common people, to paint them and their concerns. And who among the common people has not gazed upward wishing to decipher the mysteries of the sky? “Looking at the stars always makes me dream,” he wrote, “Why, I ask myself, shouldn’t the shining dots of the sky be as accessible as the black dots on the map of France?” Like others throughout the ages, he sought solace in the stars’ mysterious light and viewed them as symbols of hope. “Just as we take the train to get to Tarascon or Rouen, we take death to reach a star.”

The stars, and their influence on human life― domain of the scientist, let alone the lover and the poet―have roots in antiquity and were examined long before van Gogh swirled them down to earth for all to see. In the 14th century, Italian physicians ascribed a mysterious illness often turned epidemic to the adverse influence of the stars and called it *influentia*. The term influenza was first used in English in 1743 during an outbreak of the disease in Europe. Despite our continued inability to prevent its global spread, we have learned since that viruses are the culprits and that influenza has less to do with ethereal substances emanating from the stars and more with tiny droplets shared generously between patrons under the café awning and in other gathering places. We are still just as intrigued with the stars and van Gogh’s interpretations. And we have astronomy, as the Bard put it, “But not to tell of good or evil luck, / Of plagues, of dearths, or seasons’ quality.”
